# An Essay on the Nature, Causes, and Treatment of Deafness; and on the Diseases and Obstructions of the Cavity of the Tympanum, and of the Eustachian Tube, as Causes of Deafness; and a New Method of Treating Them by Medicated Vapour. With a List of Cases

**Published:** 1845-01

**Authors:** 


ART. III.—1. *An Essay on the Nature, Causes, and Treatment of Deafness; and on the Diseases and Obstructions of the Cavity of the Tympanum, and of the Eustachian Tube, as causes of Deafness; and a new method of treating them by Medicated Vapour. With a List of Cases.* By D. CRONIN, Surgeon.—*London,* 1840. 12mo, pp. 114.

2. *Observations on the Efficacy of Traction in the Cure of Consumption and Asthma. With authentic Cases.* By D. CRONIN, Surgeon to the Free Dispensary for Asthma, Consumption, and Diseases of the Heart, 94, St. Martin's Lane.—*London,* 1843. 12mo, pp. 70.

3. *Observations on the Preparation and Administration of Arnica Montana, or Leopard's Bane, and Scrophularia Nodosa, in the Cure of Nervous Deafness and Palpitation of the Heart.* By D. CRONIN, M.D., Physician to the Strand Dispensary for Consumption, Asthma, and Diseases of the Heart.—*London,* 1844. 12mo, pp. 52.

IN our last Number, in speaking of certain professed curers of consumption, we gently insinuated that the author of the above works might be ignorant of the natural history of this disease, and might, in so far, be regarded as a "blockhead." We have, however, since found out our mistake; and we hereby beg to apologize to our readers for the employment of an epithet, which a more careful perusal of his works has shown us to be inapplicable to him. On the contrary, we regard Mr. Dr. Cronin as a very clever fellow indeed; but, having been wrong once, we are somewhat timid in fixing on the precise epithet which ought to be substituted for the obnoxious one here repudiated. Perhaps our readers may kindly assist us in the delicate task; and in order that they may have some data to go upon, we will present them with a single quotation from each of Mr. Dr. Cronin's treatises.

"By a peculiar combination I have been able to produce a fluid, and which for the present I shall call '*Acoustic*' liquid or fluid, which, when dropped into the ear, restores the hearing, in a time proportioned, in some degree, to the duration and severity of the disease. In many cases, two or three applications alone have been sufficient to produce a most obvious amendment, or even complete cure: in others a longer perseverance in the application of the fluid has been necessary. However, but few cases, unless indeed a total disorganization of the auditory apparatus has been effected, have resisted a fortnight or three weeks' application." (Essay on Deafness, p. 57.)

"Is Consumption curable, or is it not? For the information, and perhaps for the consolation, of the reader, I declare positively and unequivocally that it is in most instances curable by my method; and I have, within the last two years, by the simple means presently to be described, cured from seven to eight hundred well-marked cases of Consumption, many of which had been treated and given over by men of the very first professional eminence, both in London and the provinces." (Observations on Consumption, &c. pp. 9-10.)

"This tincture requires some care in its preparation, in order to ensure its full benefit. I have given the formula to Mr. A. Molton, No. 113, Strand, Apothecary to the 'Dispensary for Consumption and Diseases of the Heart,' from whom it may be had in a most efficient state........ I mix three drops of the tincture with an equal proportion of water, and by means of a simple tubular apparatus, convey it (vaporized) into the ear. The effect is astonishing, and the patients themselves express their great surprise at its efficacy, and the relief afforded. Some, and even apparently the worst cases, hear almost instantaneously, and can enter into, and keep up a conversation for a considerable time—what, perhaps had not occurred to them for years before. (Observations on Arnica, pp. 18-19.)

"Reflecting upon the effects of Arnica, as a tranquillizer of the nervous system, as noticed in a previous part of this Essay, I was resolved to test its powers and its virtues in palpitation, and I was much gratified to find that I had appealed to a very active and efficient remedy. In many cases, the history of which I have hereunto appended, I had the good fortune not only to afford considerable relief, even to the most hopeless, but farther, in by far the majority, to effect a complete cure." (Ibid., pp. 37-8.)

